# Supplementary material for: Distinct HAND2/HAND2-AS1 Expression Levels May Fine-Tune Mesenchymal and Epithelial Cell Plasticity of Human Mesenchymal Stem Cells
Source: Int J Mol Sci. 2023 Nov 20;24(22):16546. doi: 10.3390/ijms242216546 (PMC10672054; doi:10.3390/ijms242216546)
Supplement: Supplementary file 1 [file ijms-24-16546-s001.zip › ijms-2651859-supplementary.pdf]

Distinct HAND2/HAND2-AS1 Expression Levels May Fine-tune Mesenchymal and Epithelial Cell Plasticity of Human Mesenchymal Stem Cells

Racheli Vazana-Netzarim [1], Yishay Elmalem [2], Shachar Sofer [2], Hod Bruck [2], Naama Danino[1], & Udi Sarig [1,2,*]

[1] The Dr. Miriam and Sheldon Adelson School of Medicine, Department of Morphological Sciences and Teratology, Ariel University.

[2] Department of Chemical Engineering and Biotechnology, Faculty of Engineering, Ariel University.

* Corresponding Author:

Dr. Udi Sarig, PhD

Head–The Laboratory for Advanced Tissue Technologies (LATi-Tech)

Department of Morphological Sciences and Teratology

Dr. Miriam and Sheldon Adelson School of Medicine

Ariel University,

Ariel, 4070000

Israel

Email: udis@ariel.ac.il

Supplementary Information Online

Supplementary Table S1: Gene array related to endothelial cell phenotypes

Position	Unigene	GeneBank	Symbol	Description	Gene Name
A01	Hs.654434	NM_000789	ACE	Angiotensin I converting enzyme	ACE1, CD143, DCP, DCP1, MGC26566, MVCD3
A02	Hs.404914	NM_003183	ADAM17	ADAM metallopeptidase domain 17	ADAM18, CD156B, CSVP, MGC71942, TACE
A03	Hs.19383	NM_000029	AGT	Angiotensinogen	ANHU, FLJ92595, FLJ97926, SERPINA8
A04	Hs.728754	NM_031850	AGTR1	Angiotensin II receptor, type 1	AG2S, AGTR1A, AGTR1B, AT1, AT1B, AT1R, AT2R1
A05	Hs.89499	NM_000698	ALOX5	Arachidonate 5-lipoxygenase	5-LO, 5-LOX, 5LPG, LOG5, MGC163204
A06	Hs.369675	NM_001146	ANGPT1	Angiopoietin 1	AGP1, AGPT, ANG1
A07	Hs.480653	NM_001154	ANXA5	Annexin A5	ANX5, ENX2, PP4
A08	Hs.654439	NM_000041	APOE	Apolipoprotein E	AD2, LDLCQ5, LPG, MGC1571
A09	Hs.624291	NM_004324	BAX	BCL2-associated X protein	BCL2L4
A10	Hs.150749	NM_000633	BCL2	B-cell CLL/lymphoma 2	Bcl-2
A11	Hs.516966	NM_138578	BCL2L1	BCL2-like 1	BCL-XL, S, BCL2L, BCLX, BCLXL, BCLXS, Bcl-X
A12	Hs.37058	NM_001741	CALCA	Calcitonin-related polypeptide alpha	CALC1, CGRP, CGRP-I, CGRP1, CT, KC, MGC126648
B01	Hs.2490	NM_033292	CASP1	Caspase 1, apoptosis-related cysteine peptidase	ICE, IL1BC, P45
B02	Hs.141125	NM_004346	CASP3	Caspase 3, apoptosis-related cysteine peptidase	CPP32, CPP32B, SCA-1

Position	Unigene	GeneBank	Symbol	Description	Gene Name
B03	Hs.74034	NM_001753	CAV1	Caveolin 1, caveolae protein, 22kDa	BSCL3, CGL3, MSTP085, VIP21
B04	Hs.303649	NM_002982	CCL2	Chemokine (C-C motif) ligand 2	GDCF-2, HC11, HSMCR30, MCAF, MCP-1, MCP1,
B05	Hs.514821	NM_002985	CCL5	Chemokine (C-C motif) ligand 5	D17S136E, MGC17164, RANTES, SCYA5, SISd, TCP228
B06	Hs.76206	NM_001795	CDH5	Cadherin 5, type 2 (vascular endothelium)	7B4, CD144, FLJ17376
B07	Hs.390736	NM_003879	CFLAR	CASP8 and FADD-like apoptosis regulator	CASH, CASP8AP1, CLARP, Casper, FLAME, FLAME-1, FLAME1, FLIP,
B08	Hs.517356	NM_030582	COL18A1	Collagen, type XVIII, alpha 1	FLJ27325, FLJ34914, KNO, KNO1, KS, MGC74745
B09	Hs.531668	NM_002996	CX3CL1	Chemokine (C-X3-C motif) ligand 1	ABCD-3, C3Xkine, CXC3, CXC3C, NTN, NTT,
B10	Hs.511899	NM_001955	EDN1	Endothelin 1	ET1, HDLCQ7, PPET1
B11	Hs.1407	NM_001956	EDN2	Endothelin 2	ET2, PPET2
B12	Hs.183713	NM_001957	EDNRA	Endothelin receptor type A	ETA, ETAR, ETRA
C01	Hs.76753	NM_000118	ENG	Endoglin	CD105, END, FLJ41744, HHT1, ORW, ORW1
C02	Hs.482562	NM_001992	F2R	Coagulation factor II (thrombin) receptor	CF2R, HTR, PAR-1, PAR1, TR
C03	Hs.62192	NM_001993	F3	Coagulation factor III (thromboplastin, tissue factor)	CD142, FLJ17960, TF, TFA
C04	Hs.244139	NM_000043	FAS	Fas (TNF receptor superfamily, member 6)	ALPS1A, APO-1, APT1, CD95, FAS1, FASTM, TNFRSF6

Position	Unigene	GeneBank	Symbol	Description	Gene Name
C05	Hs.2007	NM_000639	FASLG	Fas ligand (TNF superfamily, member 6)	APT1LG1, CD178, CD95-L, CD95L, FASL, TNFSF6
C06	Hs.483635	NM_000800	FGF1	Fibroblast growth factor 1 (acidic)	AFGF, ECGF, ECGF-beta, ECGFA, ECGFB, FGF-alpha
C07	Hs.284244	NM_002006	FGF2	Fibroblast growth factor 2 (basic)	BFGF, FGFB, HBGF-2
C08	Hs.654360	NM_002019	FLT1	Fms-related tyrosine kinase 1	FLT, VEGFR1
C09	Hs.203717	NM_002026	FN1	Fibronectin 1	CIG, DKFZp686F10164, DKFZp686H0342, DKFZp686I1370
C10	Hs.597216	NM_001530	HIF1A	Hypoxia inducible factor 1, alpha subunit	HIF-1alpha, HIF1, HIF1-ALPHA, MOP1, PASD8, bHLHe78
C11	Hs.517581	NM_002133	HMOX1	Heme oxygenase (decycling) 1	HO-1, HSP32, bK286B10
C12	Hs.643447	NM_000201	ICAM1	Intercellular adhesion molecule 1	BB2, CD54, P3.58
D01	Hs.467304	NM_000641	IL11	Interleukin 11	AGIF, IL-11
D02	Hs.126256	NM_000576	IL1B	Interleukin 1, beta	IL-1, IL1-BETA, IL1F2
D03	Hs.694	NM_000588	IL3	Interleukin 3 (colony-stimulating factor, multiple)	IL-3, MCGF, MGC79398, MGC79399, MULTI-CSF
D04	Hs.654458	NM_000600	IL6	Interleukin 6 (interferon, beta 2)	BSF2, HGF, HSF, IFNB2, IL-6
D05	Hs.591873	NM_000880	IL7	Interleukin 7	IL-7
D06	Hs.505654	NM_002205	ITGA5	Integrin, alpha 5 (fibronectin receptor, alpha polypeptide)	CD49e, FNRA, VLA5A
D07	Hs.436873	NM_002210	ITGAV	Integrin, alpha V (vitronectin receptor, alpha	CD51, DKFZp686A08142, MSK8, VNRA

Position	Unigene	GeneBank	Symbol	Description	Gene Name
				polypeptide, antigen CD51)	
D08	Hs.643813	NM_002211	ITGB1	Integrin, beta 1	CD29, FNRB, GPIIA, MDF2, MSK12, VLA-BETA, VLAB
D09	Hs.218040	NM_000212	ITGB3	Integrin, beta 3 (platelet glycoprotein IIIa, antigen CD61)	CD61, GP3A, GPIIIa
D10	Hs.479756	NM_002253	KDR	Kinase insert domain receptor	CD309, FLK1, VEGFR, VEGFR2
D11	Hs.479754	NM_000222	KIT	V-kit Hardy-Zuckerman 4 feline sarcoma viral oncogene homolog	C-Kit, CD117, PBT, SCFR
D12	Hs.171995	NM_001648	KLK3	Kallikrein-related peptidase 3	APS, KLK2A1, PSA, hK3
E01	Hs.83169	NM_002421	MMP1	Matrix metallopeptidase 1	CLG, CLGN
E02	Hs.513617	NM_004530	MMP2	Matrix metallopeptidase 2	CLG4, CLG4A, MMP-II, MONA, TBE-1
E03	Hs.297413	NM_004994	MMP9	Matrix metallopeptidase 9	CLG4B, GELB, MANDP2, MMP-9
E04	Hs.707978	NM_000603	NOS3	Nitric oxide synthase 3 (endothelial cell)	ECNOS, eNOS
E05	Hs.219140	NM_002521	NPPB	Natriuretic peptide B	BNP
E06	Hs.490330	NM_000906	NPR1	Natriuretic peptide receptor A/guanylate cyclase A	ANPRA, ANPa, GUC2A, GUCY2A, NPRA
E07	Hs.592605	NM_002538	OCLN	Occludin	BLCPMG, FLJ08163, FLJ18079, FLJ77961, FLJ94056, MGC34277

Position	Unigene	GeneBank	Symbol	Description	Gene Name
E08	Hs.74615	NM_006206	PDGFRA	Platelet-derived growth factor receptor, alpha polypeptide	CD140A, MGC74795, PDGFR2, RHEPDGFRA
E09	Hs.514412	NM_000442	PECAM1	Platelet/endothelial cell adhesion molecule	CD31, FLJ34100, FLJ58394, PECAM-1
E10	Hs.81564	NM_002619	PF4	Platelet factor 4	CXCL4, MGC138298, SCYB4
E11	Hs.252820	NM_002632	PGF	Placental growth factor	D12S1900, PGFL, PLGF, PlGF-2, SHGC-10760
E12	Hs.491582	NM_000930	PLAT	Plasminogen activator, tissue	DKFZp686I03148, T-PA, TPA
F01	Hs.77274	NM_002658	PLAU	Plasminogen activator, urokinase	ATF, UPA, URK, u-PA
F02	Hs.143436	NM_000301	PLG	Plasminogen	DKFZp779M0222
F03	Hs.647450	NM_006404	PROCR	Protein C receptor, endothelial	CCCA, CCD41, CD201, EPCR, MGC23024, bA42O4.2
F04	Hs.302085	NM_000961	PTGIS	Prostaglandin I2 (prostacyclin) synthase	CYP8, CYP8A1, MGC126858, MGC126860, PGIS, PTGI
F05	Hs.196384	NM_000963	PTGS2	Prostaglandin-endoperoxide synthase 2	COX-2, COX2, GRIPGHS, PGG, HS, PGHS-2, PHS-2, hCox-2
F06	Hs.395482	NM_005607	PTK2	PTK2 protein tyrosine kinase 2	FADK, FAK, FAK1, FRNK, pp125FAK
F07	Hs.89546	NM_000450	SELE	Selectin E	CD62E, ELAM, ELAM1, ESEL, LECAM2
F08	Hs.728756	NM_000655	SELL	Selectin L	CD62L, LAM1, LECAM1, LEU8, LNHR, LSEL, LYAM1, PLNHR, TQ1
F09	Hs.591014	NM_003006	SELPLG	Selectin P ligand	CD162, CLA, PSGL-1, PSGL1

Position	Unigene	GeneBank	Symbol	Description	Gene Name
F10	Hs.414795	NM_000602	SERPINE1	Serpin peptidase inhibitor, clade E	PAI, PAI-1, PAI1, PLANH1
F11	Hs.443914	NM_000454	SOD1	Superoxide dismutase 1, soluble	ALS, ALS1, IPOA, SOD, hSod1, homodimer
F12	Hs.68061	NM_021972	SPHK1	Sphingosine kinase 1	SPHK
G01	Hs.89640	NM_000459	TEK	TEK tyrosine kinase, endothelial	CD202B, TIE-2, TIE2, VMCM, VMCM1
G02	Hs.516578	NM_006287	TFPI	Tissue factor pathway inhibitor	EPI, LACI, TFI, TFPI1
G03	Hs.645227	NM_000660	TGFB1	Transforming growth factor, beta 1	CED, DPD1, LAP, TGFB, TGFbeta
G04	Hs.2030	NM_000361	THBD	Thrombomodulin	AHUS6, BDCA3, CD141, THRM, TM
G05	Hs.164226	NM_003246	THBS1	Thrombospondin 1	THBS, THBS-1, TSP, TSP-1, TSP1
G06	Hs.522632	NM_003254	TIMP1	TIMP metallopeptidase inhibitor 1	CLGI, EPA, EPO, FLJ90373, HCI, TIMP
G07	Hs.241570	NM_000594	TNF	Tumor necrosis factor	DIF, TNF-alpha, TNFA, TNFSF2
G08	Hs.478275	NM_003810	TNFSF10	Tumor necrosis factor (ligand) superfamily, member 10	APO2L, CD253, TL2, TRAIL
G09	Hs.592212	NM_001953	TYMP	Thymidine phosphorylase	ECGF, ECGF1, MEDPS1, MNGIE, MTDPS1, PDECGF, TP, hPD-ECGF
G10	Hs.109225	NM_001078	VCAM1	Vascular cell adhesion molecule 1	CD106, DKFZp779G2333, INCAM-100, MGC99561
G11	Hs.73793	NM_003376	VEGFA	Vascular endothelial growth factor A	MGC70609, MVCD1, VEGF, VPF

Position	Unigene	GeneBank	Symbol	Description	Gene Name
G12	Hs.440848	NM_000552	VWF	Von Willebrand factor	F8VWF, VWD

Supplementary Table S2: Gene array related to cardiomyocyte phenotypes

Unigene	GeneBank	Symbol	Description	Gene Name
Hs.498178	NM_001103	ACTN2	Actinin, alpha 2	CMD1AA
Hs.99913	NM_000684	ADRB1	Adrenergic, beta-1-, receptor	ADRB1R, B1AR, BETA1AR, RHR
Hs.334347	NM_001824	CKM	Creatine kinase, muscle	CKMM, M-CK
Hs.594952	NM_001927	DES	Desmin	CMD1I, CSM1, CSM2, FLJ12025, FLJ39719, FLJ41013, FLJ41793
Hs.243987	NM_002052	GATA4	GATA binding protein 4	MGC126629
Hs.388245	NM_021973	HAND2	Heart and neural crest derivatives expressed 2	DHAND2, FLJ16260, Hed, MGC125303, MGC125304, Thing2, bHLHa26, dHand
Hs.95162	NM_000218	KCNQ1	Potassium voltage-gated channel, KQT-like subfamily, member 1	ATFB1, ATFB3, FLJ26167, JLNS1, KCNA8, KCNA9, KVLQT1, Kv1.9, Kv7.1, LQT, LQT1, RWS, SQT2, WRS
Hs.517586	NM_005368	MB	Myoglobin	MGC13548, PVALB
Hs.929	NM_000257	MYH7	Myosin, heavy chain 7, cardiac muscle, beta	CMD1S, CMH1, DKFZp451F047, MGC138376, MGC138378, MPD1, MYHCB, SPMD, SPMM
Hs.75535	NM_000432	MYL2	Myosin, light chain 2, regulatory, cardiac, slow	CMH10, DKFZp779C0562, MLC2
Hs.517939	NM_000258	MYL3	Myosin, light chain 3, alkali; ventricular, skeletal, slow	CMH8, MLC1SB, MLC1V, VLC1

Unigene	GeneBank	Symbol	Description	Gene Name
Hs.75636	NM_021223	MYL7	Myosin, light chain 7, regulatory	MYL2A, MYLC2A
Hs.54473	NM_004387	NKX2-5	NK2 homeobox 5	CHNG5, CSX, CSX1, FLJ52202, FLJ97166, FLJ97195, FLJ97197, FLJ99536, NKX2.5, NKX2E, NKX4-1
Hs.75640	NM_006172	NPPA	Natriuretic peptide A	ANF, ANP, ATFB6, CDD-ANF, PND
Hs.170839	NM_002667	PLN	Phospholamban	CMD1P, PLB
Hs.109514	NM_001035	RYR2	Ryanodine receptor 2 (cardiac)	ARVC2, ARVD2, RYR-2, VTSIP
Hs.728911	NM_021097	SLC8A1	Solute carrier family 8 (sodium/calcium exchanger), member 1	DKFZp779F0871, FLJ37694, FLJ43417, MGC119581, NCX1
Hs.644596	NM_000363	TNNI3	Troponin I type 3 (cardiac)	CMD1FF, CMD2A, CMH7, MGC116817, RCM1, TNNC1, cTnI
Hs.533613	NM_000364	TNNT2	Troponin T type 2 (cardiac)	CMH2, CMPD2, LVNC6, MGC3889, RCM3, TnTC, cTnT
Hs.591847	NM_000662	NAT1	N-acetyltransferase 1 (arylamine N-acetyltransferase)	AAC1, MNAT, NAT-1, NATI
Hs.592355	NM_002046	GAPDH	Glyceraldehyde-3-phosphate dehydrogenase	G3PD, GAPD, MGC88685

All gene arrays were performed in a 96-well plate format, with at least n=3 biological sample repetitions. Fold regulation ($2^{-\Delta\Delta(Ct)}$) expression values (following interpolate calibration and normalization to house-keeping genes and plate controls) were obtained using Qiagen web-based software based on the company's recommended analysis pipeline. The results (**Supplementary Table S3**) indicated an initially high HAND2 spike

in the cardiomyocyte arrays (Day 4, 2409-fold regulation compared to plate controls), followed by gradually increased expression of endothelial cell markers at longer time points (for clarity, non-significant results are not reported).

Supplementary Table S3: mRNA RT-qPCR fold regulation of hMSC expression for significantly up-regulated endothelial and cardiomyocyte genes

	Gene	Fold Regulation Day 4	Day 23	Day 30
ENDOTHELIAL	APOE	75.24	157.1	288.44
	CASP1	3.05	2.55	8.97
	CCL2	21.32	6.03	16.62
	CCL5	2.24	6.08	-1.52
	COL18A1	15.97	1.69	2.68
	ICAM1	22.86	11.81	17.87
	IL11	11.3	1.65	3.59
	IL1b	125.27	8.55	34.36
	IL6	14.51	16.98	17.56
	IL7	7.37	7.69	11.69
	KDR	13.72	10.28	5.51
	MMP9	12.43	93.29	12.22
	PECAM1*	16.68	17.87	22.05
	PF4	6.03	-1.3	4.81
	PTGS2	21.6	1.42	1.51
	SLC8A1	-5.84	-3.58	-5.39
	THBD	6.34	15.62	3.39
	TIMP1	5.68	2.7	6.93
	TNSF10	-1.09	8.1	6.43
	TYMP	6.38	2.78	3.88
	VCAM1	42.79	96.45	177.09
	VEGFA	5.53	5.24	2.93
	vFW	5.44	3.63	3.97
CARDIAC	DES	29.18	2.92	4.83
	HAND2*	2409.29	9.51	1.64
	MYL3	1.31	1.84	10.64

*Red highlighted gene expression was also verified at the protein level in Figure 1.

CRISPR-Cas9 editing

CRISPR-Cas9 gene editing was outsourced to the Animal Gene Editing Laboratory (AGEL) at the Biological Resource Center (BRC) at the Agency for Science Technology & Research (A*Star), Singapore. Briefly, prior to gene editing, hMSCs were screened for diploidy and were found to be suitable for transfection. Cells were transfected (electroporation without any liposomal aids) with a pCEP4-PACdTKmchy-HAND2/HAND2AS1_1&2_2 episomal vector (**Supplementary Figure S1**) along with two flanking guide RNA (gRNA) vectors

(under a human U6 promoter) and a Cas9 vector under a CMV constitutive promoter. Successful Cas9-based editing of the flanked region activated a downstream puromycin antibiotic resistance selection gene that, following editing, became close enough to the upstream CMV promoter. Following electro-nucleofection, cells were allowed to recuperate for two days prior to antibiotic selection.

Characterization of CRISPR-Cas9 edited cells

Following selection, two types of cells appeared on the plate:

1) Type 1 cells were non-dividing, hypertrophic, and had a non-hMSC characteristic; many seemed to have a neurological-like phenotype, although this was not further verified.
2) Type 2 cells – approximately 2-3 colonies in total appeared to have a wild type-like phenotype.

Given the non-dividing nature of the type I cells, we could not subculture these plates. On Day 20, we decided to separately pick up most of the colony-forming cells while maintaining clonal identity using very short and low-volume localized trypsinization. These colonies (clones 1–3) were further sub-cultured and subsequently PCR amplified and analyzed using DNA gel electrophoresis (**Figure 1g**, clone 1, clone 2 and clone 3) compared to PCR amplifications of the original wild type (MIX WT) and the mixture of the remaining cells on the plate containing some of the colony-forming cells but mostly senescent type 2 cells (Mix MUT).

Sanger sequencing performed on the amplified PCR products confirmed that the colony clones (type I cells) were of the WT phenotype (data not shown), while MIX MUT cells comprising mostly the senescent-like cell population were positive for HAND2-HAND2-AS1 deletion (**Supplementary Fig. S2**).

Supplementary Fig. S1: CRISPR-Cas9 HAND2-HAND2-AS1 knockout plasmid map

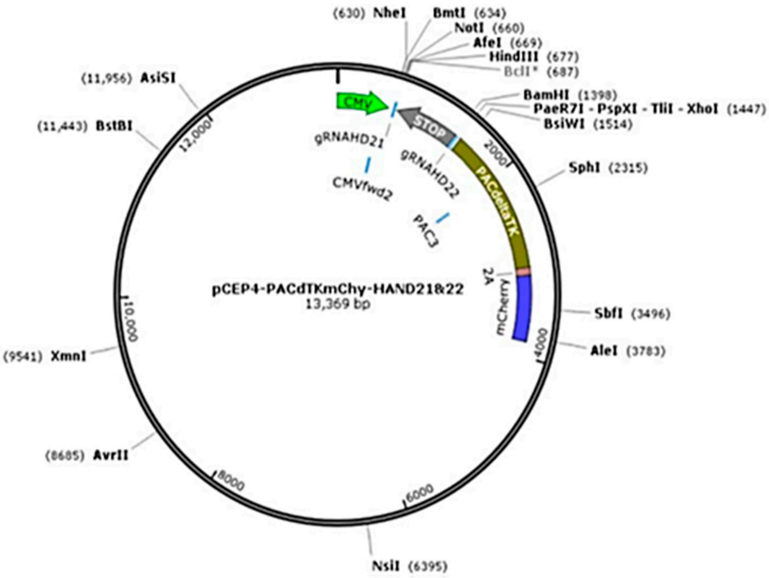

Supplementary Fig. S2: Sanger sequencing and alignment results. PCR-amplified Mix-MUT cDNA was Sanger sequenced and aligned to the original locus sequence (top). Four independent Sanger sequencing results are shown in subsequent rows. Given the length of the sequence, it appears in separate images over the next four pages.

```
             ACCAGCCTCGCGGGAGCCCCGGCACCTTTGTATGAGCACGAGAGGATTCT
                      10        20        30        40        50

humHAND2 PCR ACCAGCCTCGCGGGAGCCCCGGCACCTTTGTATGAGCACGAGAGGATTCT  50
J71_061.ab1  ACCAGCCTCGCGGGAGCCCCGGCACCTTTGTATGAGCACGAGAGGATTCT  50
J68_004.ab1  ACCAGCCTCGCGGGAGCCCCGGCACCTTTGTATGAGCACGAGAGGATTCT  50
J69_002.ab1  ACCAGCCTCGCGGGAGCCCCGGCACCTTTGTATGAGCACGAGAGGATTCT  50
J70_063.ab1  ACCAGCCTCGCGGGAGCCCCGGCACCTTTGTATGAGCACGAGAGGATTCT  50

             GCCTCCGCGCAGCAGCCCGGGAAGCAGGAGCCGAAGCGCGGGCCGTGGAG
                      60        70        80        90       100

humHAND2 PCR GCCTCCGCGCAGCAGCCCGGGAAGCAGGAGCCGAAGCGCGGGCCGTGGAG  100
J71_061.ab1  GCCTCCGCGCAGCAGCCCGGGAAGCAGGAGCCGAAGCGCGGGCCGTGGAG  100
J68_004.ab1  GCCTCCGCGCAGCAGCCCGGGAAGCAGGAGCCGAAGCGCGGGCCGTGGAG  100
J69_002.ab1  GCCTCCGCGCAGCAGCCCGGGAAGCAGGAGCCGAAGCGCGGGCCGTGGAG  100
J70_063.ab1  GCCTCCGCGCAGCAGCCCGGGAAGCAGGAGCCGAAGCGCGGGCCGTGGAG  100

             CAAGGCGGGAACCGGAGGCGGCGGCGGCGGCGGCCAGGGGCGCACGGTGC
                     110       120       130       140       150

humHAND2 PCR CAAGGCGGGAACCGGAGGCGGCGGCGGCGGCGGCCAGGGGCGCACGGTGC  150
J71_061.ab1  CAAGGCGGGAACCGGAGGCGGCGGCGGCGGCGGCCAGGGGCGCACGGTGC  150
J68_004.ab1  CAAGGCGGGAACCGGAGGCGGCGGCGGCGGCGGCCAGGGGCGCACGGTGC  150
J69_002.ab1  CAAGGCGGGAACCGGAGGCGGCGGCGGCGGCGGCCAGGGGCGCACGGTGC  150
J70_063.ab1  CAAGGCGGGAACCGGAGGCGGCGGCGGCGGCGGCCAGGGGCGCACGGTGC  150

             CAGGACCAGCTCGCCGCGCCCCATG-------------------------
                     160       170       180       190       200

humHAND2 PCR CAGGACCAGCTCGCCGCGCCCCATGGGGAGCCGGCGGCCGCAGCGCTGCT  200
J71_061.ab1  CAGGACCAGCTCGCCGCGCCCCATG-------------------------  176
J68_004.ab1  CAGGACCAGCTCGCCGCGCCCCATG-------------------------  176
J69_002.ab1  CAGGACCAGCTCGCCGCGCCCCATG-------------------------  176
J70_063.ab1  CAGGACCAGCTCGCCGCGCCCCAT--------------------------  175

             --------------------------------------------------
                     210       220       230       240       250

humHAND2 PCR GAGGCGGGCCCGGCTGGCCAGGCGGGGGACGGGGCCCGGGCTGCAGCAG  250
J71_061.ab1  --------------------------------------------------  176
J68_004.ab1  --------------------------------------------------  176
J69_002.ab1  --------------------------------------------------  176
J70_063.ab1  --------------------------------------------------  175
```

```
                   --------------------------------------------------
                            260       270       280       290       300
humHAND2 PCR       CCCCCTCTGCGGCTGCCGGGCGGGCCCGGGCGCCCGGGGGCTGGGGGGTG  300
J71_061.ab1        --------------------------------------------------  176
J68_004.ab1        --------------------------------------------------  176
J69_002.ab1        --------------------------------------------------  176
J70_063.ab1        --------------------------------------------------  175

                   --------------------------------------------------
                            310       320       330       340       350
humHAND2 PCR       GGGGGTGGGGGAGGACGCCGAGCGCTGAGGCAGGGGCCCGGGCCGAGGGC  350
J71_061.ab1        --------------------------------------------------  176
J68_004.ab1        --------------------------------------------------  176
J69_002.ab1        --------------------------------------------------  176
J70_063.ab1        --------------------------------------------------  175

                   --------------------------------------------------
                            360       370       380       390       400
humHAND2 PCR       GCGGCGGGGCTGCGCGCACGCTGGGGCGCGTGGAGGGGCGCGGAGGGCGA  400
J71_061.ab1        --------------------------------------------------  176
J68_004.ab1        --------------------------------------------------  176
J69_002.ab1        --------------------------------------------------  176
J70_063.ab1        --------------------------------------------------  175

                   --------------------------------------------------
                            410       420       430       440       450
humHAND2 PCR       AATGAGTCTGGTAGGTGGTTTTCCCCACCACCCGGTGGTGCACCACGAGG  450
J71_061.ab1        --------------------------------------------------  176
J68_004.ab1        --------------------------------------------------  176
J69_002.ab1        --------------------------------------------------  176
J70_063.ab1        --------------------------------------------------  175

                   --------------------------------------------------
                            460       470       480       490       500
humHAND2 PCR       GCTACCCGTTTGCCGCCGCCGCCGCCGCAGCTGCCGCCGCCGCCGCCAGC  500
J71_061.ab1        --------------------------------------------------  176
J68_004.ab1        --------------------------------------------------  176
J69_002.ab1        --------------------------------------------------  176
J70_063.ab1        --------------------------------------------------  175
```

```
              - - - - - - - - - - - - - - - - - - - - - - - - - - - - - - - - - - - - - - - - - - - - - - - - - -
                                510                 520                 530                 540                 550
humHAND2 PCR  C G C T G C A G C C A T G A G G A G A A C C C C T A C T T C C A T G G C T G G C T C A T C G G C C A  550
J71_061.ab1   - - - - - - - - - - - - - - - - - - - - - - - - - - - - - - - - - - - - - - - - - - - - - - - - - -  176
J68_004.ab1   - - - - - - - - - - - - - - - - - - - - - - - - - - - - - - - - - - - - - - - - - - - - - - - - - -  176
J69_002.ab1   - - - - - - - - - - - - - - - - - - - - - - - - - - - - - - - - - - - - - - - - - - - - - - - - - -  176
J70_063.ab1   - - - - - - - - - - - - - - - - - - - - - - - - - - - - - - - - - - - - - - - - - - - - - - - - - -  175
```

```
              - - - - - - - - - - - - - - - - - - - - - - - - - - - - - - - - - - - - - - - - - - - - - - - - - -
                                560                 570                 580                 590                 600
humHAND2 PCR  C C C C G A G A T G T C G C C C C C C G A C T A C A G C A T G G C C C T G T C C T A C A G C C C C G  600
J71_061.ab1   - - - - - - - - - - - - - - - - - - - - - - - - - - - - - - - - - - - - - - - - - - - - - - - - - -  176
J68_004.ab1   - - - - - - - - - - - - - - - - - - - - - - - - - - - - - - - - - - - - - - - - - - - - - - - - - -  176
J69_002.ab1   - - - - - - - - - - - - - - - - - - - - - - - - - - - - - - - - - - - - - - - - - - - - - - - - - -  176
J70_063.ab1   - - - - - - - - - - - - - - - - - - - - - - - - - - - - - - - - - - - - - - - - - - - - - - - - - -  175
```

```
              - - - - - - - - - - - - - - - - - - - - - - - - - - - - - - - - - - - - - - - - - - - - - - - - - -
                                610                 620                 630                 640                 650
humHAND2 PCR  A G T A T G C C A G C G G C G C C G C C G G C C T G G A C C A C T C C C A T T A C G G G G G G G T G  650
J71_061.ab1   - - - - - - - - - - - - - - - - - - - - - - - - - - - - - - - - - - - - - - - - - - - - - - - - - -  176
J68_004.ab1   - - - - - - - - - - - - - - - - - - - - - - - - - - - - - - - - - - - - - - - - - - - - - - - - - -  176
J69_002.ab1   - - - - - - - - - - - - - - - - - - - - - - - - - - - - - - - - - - - - - - - - - - - - - - - - - -  176
J70_063.ab1   - - - - - - - - - - - - - - - - - - - - - - - - - - - - - - - - - - - - - - - - - - - - - - - - - -  175
```

```
              - - - - - - - - - - - - - - - - - - - - - - - - - - - - - - - - - - - - - - - - - - - - - - - - - -
                                660                 670                 680                 690                 700
humHAND2 PCR  C C G C C G G G C G C C G G G C C C C C G G G C C T G G G G G G G C C G C G C C C G G T G A A G C G  700
J71_061.ab1   - - - - - - - - - - - - - - - - - - - - - - - - - - - - - - - - - - - - - - - - - - - - - - - - - -  176
J68_004.ab1   - - - - - - - - - - - - - - - - - - - - - - - - - - - - - - - - - - - - - - - - - - - - - - - - - -  176
J69_002.ab1   - - - - - - - - - - - - - - - - - - - - - - - - - - - - - - - - - - - - - - - - - - - - - - - - - -  176
J70_063.ab1   - - - - - - - - - - - - - - - - - - - - - - - - - - - - - - - - - - - - - - - - - - - - - - - - - -  175
```

```
              - - - - - - - - - - - - - - - - - - - - - - - - - - - - - - - - - - - - - - - - - - - - - - - - - -
                                710                 720                 730                 740                 750
humHAND2 PCR  C C G A G G C A C C G C C A A C C G C A A G G A G C G G C G C A G G A C T C A G A G C A T C A A C A  750
J71_061.ab1   - - - - - - - - - - - - - - - - - - - - - - - - - - - - - - - - - - - - - - - - - - - - - - - - - -  176
J68_004.ab1   - - - - - - - - - - - - - - - - - - - - - - - - - - - - - - - - - - - - - - - - - - - - - - - - - -  176
J69_002.ab1   - - - - - - - - - - - - - - - - - - - - - - - - - - - - - - - - - - - - - - - - - - - - - - - - - -  176
J70_063.ab1   - - - - - - - - - - - - - - - - - - - - - - - - - - - - - - - - - - - - - - - - - - - - - - - - - -  175
```

Supplementary Information

Alignment Workspace of Untitled J. Hein (Weighted) Page 4
Thursday, 05 October, 2017 6:32 PM

```
              --------------------------------------------------
                                 760       770       780       790       800
humHAND2 PCR  GCGCCTTCGCCGAACTGCGCGAGTGCATCCCCAACGTACCCGCCGACACC  800
J71_061.ab1   --------------------------------------------------  176
J68_004.ab1   --------------------------------------------------  176
J69_002.ab1   --------------------------------------------------  176
J70_063.ab1   --------------------------------------------------  175

              --------------------------------------------------
                                 810       820       830       840       850
humHAND2 PCR  AAACTCTCCAAAATCAAGACCCTGCGCCTGGCCACCAGCTACATCGCCTA  850
J71_061.ab1   --------------------------------------------------  176
J68_004.ab1   --------------------------------------------------  176
J69_002.ab1   --------------------------------------------------  176
J70_063.ab1   --------------------------------------------------  175

              ---------------------------------------AGGCGGAGGCC
                                 860       870       880       890       900
humHAND2 PCR  CCTCATGGACCTGCTGGCCAAGGACGACCAGAATGGCG-AGGCGGAGGCC  899
J71_061.ab1   ---------------------------------------AGGCGGAGGCC  186
J68_004.ab1   --------------------------------------CAGGCGGAGGCC  187
J69_002.ab1   --------------------------------------CAGGCGGAGGCC  187
J70_063.ab1   ---------------------------------------AGGCGGAGGCC  185

              TTCAAGGCAGAGATCAAGAAGACCGACGTGAAAGAGGAGAAGAGGAAGAA
                                 910       920       930       940       950
humHAND2 PCR  TTCAAGGCAGAGATCAAGAAGACCGACGTGAAAGAGGAGAAGAGGAAGAA  949
J71_061.ab1   TTCAAGGCAGAGATCAAGAAGACCGACGTGAAAGAGGAGAAGAGGAAGAA  236
J68_004.ab1   TTCAAGGCAGAGATCAAGAAGACCGACGTGAAAGAG-AGAAGAGGAAGAA  236
J69_002.ab1   TTCAAGGCAGAGATCAAGAAGACCGACGTGAAAGAGGAGAAGAGGAAGAA  237
J70_063.ab1   TTCAAGGCAGAGATCAAGAAGACCGACGTGAAAGAGGAGAAGAGGAAGAA  235

              GGAGCTGGTCAGTACCAGGGGGCGGCA
                                 960       970
humHAND2 PCR  GGAGCTGGTCAGTACCAGGGGGCGGCA  976
J71_061.ab1   GGAGCTGGTCAGTACCAGGGGGCGGCA  263
J68_004.ab1   GGAGCTGGTCAGTACCAGGGGGCGGCA  263
J69_002.ab1   GGAGCTGGTCAGTACCAGGGGGCGGCA  264
J70_063.ab1   GGAGCTGGTCAGTACCAGGGGGCGGCA  262
```

Study limitations: During the reviewing process, concerns were raised by one of the reviewers relating to the lack of appropriate protein level validation of the HAND2 gene editing KO study and the absence of downstream analysis or experimental work to substantiate the initial observation. Collectively, that reviewer claimed that this study design is 'fundamentally flawed' and its scientific conclusions should be taken with care. While we agree that additional work is required to validate and study the suggested mechanism based on the findings reported herein, we believe that

disseminating this work, including the accompanying criticism of it, is the only way to move forward. In particular, this is the case given the potential significance of our findings for many related fields involving hMSCs and MET-related processes in disease and therapy.
